# Energy-Efficient Online Resource Management and Allocation Optimization in Multi-User Multi-Task Mobile-Edge Computing Systems with Hybrid Energy Harvesting

**DOI:** 10.3390/s18093140

**Published:** 2018-09-17

**Authors:** Heng Zhang, Zhigang Chen, Jia Wu, Yiqing Deng, Yutong Xiao, Kanghuai Liu, Mingxuan Li

**Affiliations:** 1 School of Software, Central South University, Changsha 410075, China; zhangheng1018@csu.edu.cn (H.Z.); dengyiqin@csu.edu.cn (Y.D.); tdxytcn@gmail.com (Y.X.); Liukanghuai@csu.edu.cn (K.L.); LiMingXuan@csu.edu.cn (M.L.); 2 “Mobile Health” Ministry of Education-China Mobile Joint Laboratory, Changsha 410075, China

**Keywords:** mobile edge computing, Internet of Things, hybrid energy harvesting, Lyapunov optimization, energy efficiency, delay

## Abstract

Mobile Edge Computing (MEC) has evolved into a promising technology that can relieve computing pressure on wireless devices (WDs) in the Internet of Things (IoT) by offloading computation tasks to the MEC server. Resource management and allocation are challenging because of the unpredictability of task arrival, wireless channel status and energy consumption. To address such a challenge, in this paper, we provide an energy-efficient joint resource management and allocation (ECM-RMA) policy to reduce time-averaged energy consumption in a multi-user multi-task MEC system with hybrid energy harvested WDs. We first formulate the time-averaged energy consumption minimization problem while the MEC system satisfied both the data queue stability constraint and energy queue stability constraint. To solve the stochastic optimization problem, we turn the problem into two deterministic sub-problems, which can be easily solved by convex optimization technique and linear programming technique. Correspondingly, we propose the ECM-RMA algorithm that does not require priori knowledge of stochastic processes such as channel states, data arrivals and green energy harvesting. Most importantly, the proposed algorithm achieves the energy consumption-delay trade-off as [O(1/V),O(V)]. *V*, as a non-negative weight, which can effectively control the energy consumption-delay performance. Finally, simulation results verify the correctness of the theoretical analysis and the effectiveness of the proposed algorithm.

## 1. Introduction

As an ubiquitous computer paradigm, the Internet of Things (IoT) has seen explosive growth in computationally intensive mobile applications such as autonomous driving, virtual reality, and interactive online games [1]. Because of size and production cost considerations, wireless devices (WDs) (e.g., sensors) in IoT systems generally carry capacity-constrained batteries and energy-saving, low-performance processors. Therefore, the development of computationally intensive applications is severely limited by resource-constrained devices [2,3]. Consequently, how to eliminate this bottleneck is a key issue in the research and development of modern Internet of Things technology.

Currently, this limitation is solved by transferring computation and storage from the resource-constrained devices to clouds. However, cloud computing resources are deployed in large data centers far from most users, resulting in communication delay and high energy consumption between users and clouds [4]. In order to solve these problems, the concept of edge and fog computing was proposed [5]. MEC (Mobile Edge Computing)is a promising technology that can overcome these challenges and allows WDs to access cloud computing services on the access point (AP) and on base station (BS) integrated the edge servers. In the MEC system, resource-constrained WDs can offload computation tasks to APs and then these tasks computed by MEC servers are deployed on the APs [6]. The edge computing platform differs from cloud computing in that the computing resources are at the edge of the network and are near the end point device. Therefore, it is possible for MEC to reduce communication delay and the bandwidth requirement of the network connection to the remote data center. In addition, based on the convenience of data processing and storage near users, some new research contents will be generated [5,7]. At present, there are many studies on the direction of computation offloading in the field of MEC.

The computation offloading technology in the MEC can effectively enhance the computation capability of WDs, but how can we provide a sustainable, stable and effective energy supply for WDs? Energy harvesting (EH) technology is emerging as a promising paradigm to solve the limited battery capacity problem. Currently, green energy has been widely used in the industry as an environmentally friendly renewable energy source [8,9,10]. There are many benefits to using green energy, but there are some problems with low energy conversion rate tightly correlated to the environmental conditions. Consequently, green energy is highly unpredictable and has instability [11]. To tackle this issue, we introduce radio frequency (RF) based on wireless power transfer (WPT). WPT can use RF energy transmitters to continuously charge the batteries of remote energy harvesting IoT devices [2,12,13]. Therefore, we use green energy supply as a primary energy source of WDs, and the backup energy source supply is wireless energy based on WPT.

In order to improve the energy efficiency and performance of WDs in an MEC system, in addition to designing an efficient computational offloading scheme, we need to consider the following issues: (i) How much transmit power should be allocated to offloading computation tasks; (ii) How long to offload tasks; and (iii) how to manage the battery of an WD to ensure the normal operation of the device? In order to solve the above problems, we focus on the joint resource management and allocation issues. The main contributions are as follows:

- We consider a multi-user multi-task MEC system with hybrid energy harvesting WDs to investigate the joint resource management and allocation problem. To solve the stochastic optimization problem, we turn the problem into two deterministic sub-problems, namely transmission optimization sub-problem and battery management sub-problems. The transmission optimization sub-problem can be designed to solve the transmission power and the transmission time of data, while the battery management sub-problem can be constructed to solve the needed wireless energy amount.
- Based on Lyapunov optimization theory and convex optimization theory, we use the proposed energy-efficient joint resource management and allocation algorithm (ECM-RMA) to solve the problem of minimizing energy consumption under delay guarantees. Without the prior knowledge, the algorithm allocates energy offloading data according to the energy harvest amount, remaining available time, amount of data to be processed and other factors at the beginning of each time slot.
- The trade-off between energy consumption and delay is [O(1/V),O(V)]. *V*, as a non-negative weight, can achieve the balance between energy consumption and task data queues.
- In the field of MEC, we first proposed the devices with a hybrid energy harvesting method that integrates green energy and wireless energy.

## 2. Related Works

Recently, the academic community has done a lot of research on resource management and allocation strategies in an MEC system. These policies mainly manage and distribute resources of local execution, comunication process, and edge servers. The scenario of resource management and allocation in MEC systems is divided into single-user MEC systems [14,15,16,17,18,19], multi-user MEC systems [20,21,22,23,24,25,26] and heterogeneous server MEC systems [27,28]. In [14], the offloading ratio, transmission power, and the CPU clock frequency are jointly optimized to minimize the latency subject to the energy consumption or minimize the energy consumption subject to the latency. In [19], Mahmoodi jointly optimizes the computational latency and energy consumption, which only based on the Markov decision processes (MDP) theory to optimize the size of the offloading data. In order to minimize the total energy consumption, an optimization strategy is proposed in [20], which has a simple threshold structure for the size of offloading data and time allocation, and is controlled by the optimization strategy. In [21], instead of controlling the size of the offloading data and the time allocation, Barbarossa minimizes the overall energy consumption by controlling the transmission power and the server CPU clock. However, the above works did not consider a real application scenario, which is resource management and allocation under multi-user and multi-task.

In order to prolong the battery life of wireless IoT devices and to reduce its manual maintenance costs, some recent works have begun to explore renewable energy supplies and wireless transfer energy supplies in MEC systems. In [29,30], researchers incorporate renewable energy into MEC. In [29], an effective resource management algorithm based on reinforcement learning is proposed by Xu and Ren. Compared with standard reinforcement learning algorithms such as Q-learing, learning speed and time performance of the proposed effective resource management algorithms are significantly improved. Maos. proposed a low-complexity online algorithm to jointly determine the offloading strategy, CPU cycle frequency and transmission power to achieve an asymptotically optimal of execution cost in [30]. In wireless powered MEC scenes, an optimized resource allocation strategy in [6] is proposed by Wang, and the total energy consumption of the AP is minimized under the user’s own computational delay constraint. In addition, in a multi-user wireless powered MEC network, in order to maximize the weighted total calculation rate, an alternating direction method of multipliers (ADMM) -based technique by jointly optimizing the computational model and transmission time allocation was proposed in [2]. However, the strong randomness of renewable energy cannot guarantee the reliability of local execution or offloading. In addition, in the wireless transfer energy powered MEC system, it will cause some energy consumption to the energy transmission end. To the best of our knowledge, it has not been proposed that the devices in an MEC system use a hybrid energy harvesting method for local computing and offloading.

In addition, the number of available sub-channels in the IoT network also have an larger impact on tasks offloading. In [1], Mao use a joint computation allocation and resource management algorithm to research the trade-off of fundamental between Energy Efficiency and delay. In order to minimize energy consumption of device while meeting delay constarint of user, Hao et al. propose a joint task offloading and caching problem in [31]. An energy-efficient dynamic offloading resource scheduling (eDors) policy is proposed by Guo to shorten task completion time and reduce energy consumption [25]. However, the above works did not consider the number of available sub-channels in an MEC system.

The remainder of this paper is as follows: Section 3 shows the system model and the problem of minimizing the average total energy consumption subject to latency is formulated. Section 4 proposes an efficient online resource management and allocation algorithm to solve the formulated problem and the performance analysis of the algorithm is given. Section 5 provides simulation results to verify the correctness of the theoretical analysis and the effectiveness of the proposed algorithm. Finally, Section 6 concludes this paper. The key mathematical notations used in this paper are listed in Table 1, and the abbreviations used in the Table 1 are defined in Table 2.

## 3. System Model

**Figure 1:**
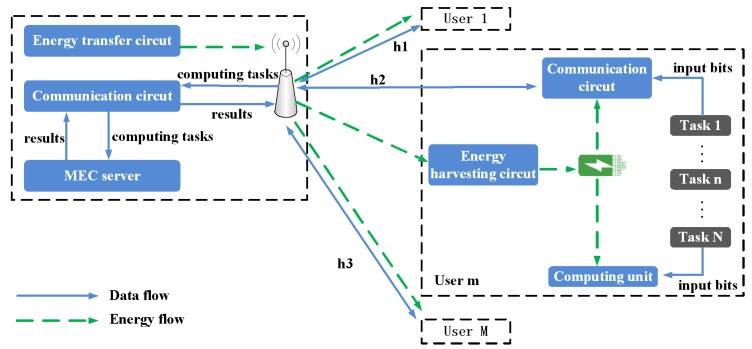
The detailed multi-user multi-task MEC (Mobile Edge Computing) system architecture when only considering IoT (Internet of Things) devices with wireless powered model.

As shown in Figure 1, we consider a multi-user multi-task MEC system with hybrid energy harvesting, which consists of an AP (integrated with an RF energy transmitter, an MEC server and a communication circuit) and *M* WDs (each WD contains an energy harvesting circuit, a rechargeable battery, a computing unit and a communication circuit). We denote the set of WDs by M = {1, 2, …, *M*} and use N = {1, 2, …, *N*} to denote the set of computation tasks of WD *m*. Each WD has a completion deadline, and $J_{m}$ denotes the completion deadline of WD *m*. Moreover, the number of available sub-channels for the WDs is denoted by *K*. We divide the total time into slots denoted by set of T = {0, 1, …,} and *t* ∈ T with slot length *T*. Within each time frame, some computationally intensive tasks can be offloaded to the AP through uplink wireless links and computed at the MEC server, while others are directly computed locally.

We assume that the AP knows the channel status information (CSI), task offloading information, and the remaining battery power of WDs, which can be obtained through feedback [20]. Using this information, at the beginning of slot *t*, the data transmission power, the transmission time and the energy need to be harvested by each device will be determined by the AP. In addition, then the AP sends these decisions to the WDs in the MEC system. Specially, we only need to focused on one time slot.

**Figure 2:**
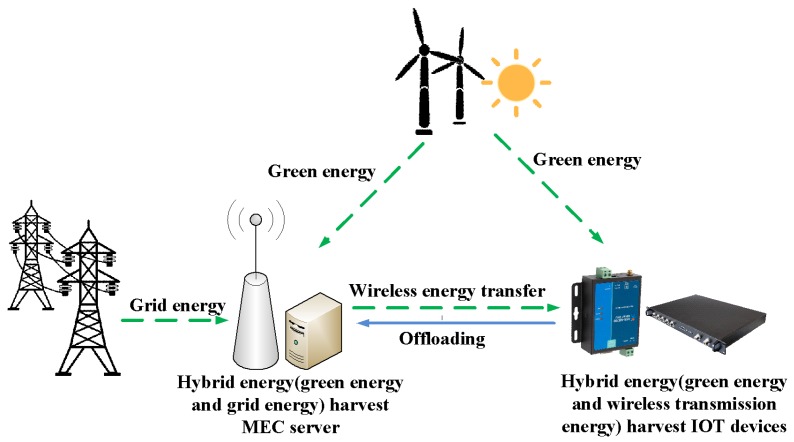
A multi-user multi-task MEC system with hybrid energy harvesting.

From Figure 2, the WDs in a hybrid energy harvesting MEC system powered by green energy from the environment and wireless transfer energy from the AP as well as the AP powered by green energy and grid energy can be seen. In detail, when a device cannot get enough energy from the environment to fill the rechargeable battery, AP in the MEC system will transmit wireless energy to fill the device battery. In addition, for the AP, green energy is used as the primary source of energy, while stable grid energy as a backup energy supply [2]. Then, the harvested energy of a WD is used by the computing unit and the communication circuit to compute tasks locally and offload tasks, respectively.

### 3.1. Communication Model

We first introduce the communication model between the WDs and the AP in an MEC system. Small-scale Rayleigh fading is followed by all wireless channels. Therefore, the wireless channel states are independent and remain unchanged during each time slot. During a time slot *t*, the uplink transmission rate between WD *m* and the AP is quantified as

(1) $$\begin{array}{c} \nu_{m}\left(t\right)\;=\;B_{m,ul}\log_{2}\left(1+\frac{p_{m}\left(t\right)h_{m,ul}\left(t\right)}{w_{0}}\right), \end{array}$$

where $B_{m,ul}$ is the uplink channel bandwidth, which is allocated to WD *m* by the AP. $p_{m}\left(t\right)$ represents the transmission power between WD *m* and the AP at time slot *t*. Moreover, $p_{m}\left(t\right)$ with the minimum value $p_{m,min}$ and the maximum value $p_{m,max}$, i.e.,

(2) $$\begin{array}{c} p_{m,min}\leq p_{m}\left(t\right)\leq p_{m,max}. \end{array}$$

$h_{m,ul}\left(t\right)$ is the uplink channel gain between WD *m* and the AP. Specially, the channel gain is determined by small-scale Rayleigh fading and the distance-dependent path loss. In addition, $w_{0}$ is the white noise power level.

At the same time, a WD can only occupy one available sub-channel. Let $\tau_{m}^{n}\left(t\right)$ denote the duration of task *n* partially offloaded from WD *m* to the AP within time slot *t*, i.e.,

(3) $$\begin{array}{c} 0\leq \tau_{m}^{n}\left(t\right)\leq T. \end{array}$$

Moreover, we define $\tau_{m}\left(t\right)=\left\{\tau_{m}^{1}\left(t\right),\tau_{m}^{2}\left(t\right),\ldots ,\tau_{m}^{N}\left(t\right)\right\}$ as the time allocated schedule of all tasks in WD *m* during slot *t*, and the total uplink transmission time of WD *m* must not exceed the available slot length, given as

(4) $$\begin{array}{c} \sum_{n\in N}\tau_{m}^{n}\left(t\right)\leq T. \end{array}$$

In addition, the total uplink transmission time of the all tasks on all devices must not exceed the sum of the time lengths of all available sub-channels, which is as follows:

(5) $$\begin{array}{c} \sum_{m\in M}\sum_{n\in N}\tau_{m}^{n}\left(t\right)\leq KT, \end{array}$$

where *K* denotes the number of available sub-channels in the MEC system, and each WD can only access an available sub-channel at one time slot due to the WDs in a IoT network being narrow-band and simple.

In this paper, we don’t consider the delay, energy consumption and packet loss of downlink transmission. This is because the size of the result returned is normally much smaller than the corresponding pre-processing task. In addition, the downlink transmission rate between the WDs and the AP is higher than the corresponding uplink transmission rate [6,31].

### 3.2. Task Computation Model

In this section, we introduce the task computation model. In practice, many computed tasks consist of multiple small procedures/components. Thus, it is necessary to perform partial computation offloading of a task. Specifically, a computed task can be divided into multiple parts where some of the parts were executed at the WD and the others were offloaded for the AP execution. Let $I_{m}^{n}\left(t\right)$ denote the data amount of computed task *n* that arrived at WD *m* at the end of time slot *t* and the WD can continuously receive data within a time slot. $I_{m}^{n}\left(t\right)$ with the rate of data arrival $C_{m}^{n}\left(t\right)$, which obeys independently and identically distributed (i.i.d) over time slots, independent between tasks, have a maximum data arrival rate $C_{m}^{n,max}\left(t\right)$ and have average arrival data rate $\lambda_{m}^{n}$. Therefore, $I_{m}^{n}\left(t\right)$ has the maximum data arrival $I_{m}^{n,max}$, i.e.,

(6) $$\begin{array}{c} 0\leq I_{m}^{n}\left(t\right)\leq I_{m}^{n,max}. \end{array}$$

Moreover, each WD allocates the same amount of data storage space $R^{D}$ for each task, and the task data is stored in its own corresponding data storage space before it is processed.

#### 3.2.1. Local Computing

Regarding the local computing, let $D_{m,l}^{n}$ denote the data amount of task *n* computed locally by the WD *m* within a time slot *t*. The CPU-cycle frequency assigned to task *n* in WD *m* is denoted by $f_{m}^{n}$, and $L_{m}$ is the CPU cycles required to compute a one bit size of data. Therefore, the local computed data amount of task *n* in WD *m* during time slot *t* can be expressed as follows:

(7) $$\begin{array}{c} D_{m,l}^{n}\;=\;T\frac{f_{m}^{n}}{L_{m}}. \end{array}$$

The corresponding energy consumption for local computation for task *n* in WD *m* during a time slot *t* is given as follows:

(8) $$\begin{array}{c} e_{m,l}^{n}\left(t\right)\;=\;\kappa {\left[f_{m}^{n}\right]}^{3}T, \end{array}$$

where *k* is the effective switched capacitance related to the chip architecture [1].

#### 3.2.2. Mobile Edge Cloud Computing

On the other hand, because of WDs not having enough computing power, some data need to be offloaded to the AP for computation. Our paper is aimed at the energy consumption of the WDs in an MEC system, so we do not consider the energy consumption generated by the MEC server.

We assume that the offloaded data amount of task *n* in WD *m* at time slot *t* is shown by

(9) $$\begin{array}{c} D_{m,c}^{n}\left(t\right)\;=\;\nu_{m}\left(t\right)\tau_{m}^{n}\left(t\right). \end{array}$$

In addition the corresponding energy consumption of WD *m* offloading the data of task *n* at time slot *t* can written as

(10) $$\begin{array}{c} e_{m,c}^{n}\left(t\right)=p_{m}\left(t\right)\tau_{m}^{n}\left(t\right). \end{array}$$

After a task is offloaded to the MEC server, the MEC server will process the task. We assume that the computation capability of the MEC server is $f_{ap}$, and the computing resource required for task *n* of WD *m* is $g_{m}^{n}$. Hence, the computation delay generated by processing task *n* of WD *m* by the MEC server can be expressed as

(11) $$\begin{array}{c} \tau_{ap}^{m,n}=\frac{g_{m}^{n}}{f_{ap}}. \end{array}$$

As mentioned before, each WD has a completion deadline. Thus, we have

(12) $$\begin{array}{c} \sum_{n\in N}\tau_{ap}^{m,n}+\sum_{t\in T}\sum_{n\in N}\tau_{m}^{n}\left(t\right)\leq J_{m}. \end{array}$$

Clearly, we have the total computed data of task *n* in WD *m* at time slot *t*, $D_{m,n}\left(t\right)$, as given by

(13) $$\begin{array}{c} D_{m}^{n}\left(t\right)=D_{m,l}^{n}\left(t\right)+D_{m,c}^{n}\left(t\right). \end{array}$$

In this paper, each task has a data backlog queue. Hence, we use $Q_{m}^{n}\left(t\right)$ to denote the backlog of the data queue at task *n* of WD *m* and it is updated over time. Based on the above analysis, the dynamics of data backlog queue at task *n* of WD *m* can easily be expressed as

(14) $$\begin{array}{c} Q_{m}^{n}\left(t+1\right)=max\left[Q_{m}^{n}\left(t\right)-D_{m}^{n}\left(t\right),0\right]+I_{m}^{n}\left(t\right). \end{array}$$

### 3.3. Energy Consumption Model

In this section, we will introduce the energy consumption model. As we know, green energy is unpredictable and unstable. In order to be environmentally friendly and improve the reliability of energy harvesting as much as possible, we use two energy sources to support the WDs in an MEC system. The one energy source is the green energy that WDs harvested from the environment, and the other one is the wireless transfer energy from the AP. In our paper, the AP will provide wireless transfer energy to support the WD when green energy can’t support the power required by a WD.

In a given time slot, the energy that WD *m* gets from the environment is denoted as $e_{m,h}$, which is a stochastic value determined by the state of environment, and $e_{m,w}$ is defined as the energy that WD *m* gets from the AP. Moreover, the energy supply from the environment is denoted by $\gamma_{m}\left(t\right)$, that is, the green energy that WD *m* can be get at time slot *t*. $\gamma_{m}\left(t\right)$ is i.i.d in different time slots, and the upper limitation of $\gamma_{m}\left(t\right)$ is $\gamma_{max}$. Since the harvested energy from the environment by any WD cannot exceed the energy supply in a time slot, the following inequality can be obtained:

(15) $$\begin{array}{c} 0\leq e_{m,h}\left(t\right)\leq \gamma_{m}\left(t\right),\forall m\in M. \end{array}$$

In addition, $e_{m,w}$ is defined as

(16) $$\begin{array}{c} e_{m,w}\left(t\right)=\mu p_{ap}\left(t\right)h_{m,dl}\left(t\right)\ell_{m}\left(t\right), \end{array}$$

where μ∈(0,1) represents the energy harvesting efficiency of energy harvesting from an AP. $p_{ap}\left(t\right)$ and $h_{m,dl}\left(t\right)$ denote the energy transmission power of the AP and the downlink channel gain, respectively. As for $\ell_{m}\left(t\right)$, it is the transmission time of wireless energy, and it satisfies $\ell_{m}\left(t\right)\leq T$. In order to avoid mutual interference caused by the common use of channels, wireless energy transfer and task offloading cannot be performed simultaneously. Thus, we use the following formula to limit:

(17) $$\begin{array}{c} \sum_{n\in N}\tau_{m}^{n}\left(t\right)+\ell_{m}\left(t\right)\leq T. \end{array}$$

Similarly, convert Equation (5) to Equation (17)

(18) $$\begin{array}{c} \sum_{m\in M}\sum_{n\in N}\tau_{m}^{n}\left(t\right)+\sum_{m\in M}\ell_{m}\left(t\right)\leq KT. \end{array}$$

Therefore, the total energy harvested by WD *m* at time slot *t* is $e_{m,hw}\left(t\right)$, i.e.,

(19) $$\begin{array}{c} e_{m,hw}\left(t\right)=e_{m,h}\left(t\right)+e_{m,w}\left(t\right),\forall m\in M. \end{array}$$

As we mentioned before, energy harvested by each WD is stored in a rechargeable battery. The energy queue of WD *m* is denoted by $E_{m}\left(t\right)$, which is energy available in the battery of WD *m*. Within time slot *t*, the total energy consumed by WD *m* for task *n* is expressed as $e_{m,total}^{n}\left(t\right)$, which consists of two parts, the local CPU energy consumption and the data offloaded energy consumption, i.e.,

(20) $$\begin{array}{c} e_{m,total}^{n}\left(t\right)=e_{m,l}^{n}\left(t\right)+e_{m,c}^{n}\left(t\right),\forall m\in M. \end{array}$$

Based on the above analysis, the dynamic energy queue of WD *m* can be expressed as

(21) $$\begin{array}{c} E_{m}\left(t+1\right)=max[E_{m}\left(t\right)-\sum_{n\in N}e_{m,total}^{n}\left(t\right),0]+e_{m,hw}\left(t\right), \end{array}$$

where $\sum_{n\in N}e_{m,total}^{n}\left(t\right)$ satisfies the following inequality, i.e.,

(22) $$\begin{array}{c} \sum_{n\in N}e_{m,total}^{n}\left(t\right)\leq E_{m}\left(t\right), \end{array}$$

since the energy consumption of the WD *m* cannot exceed the length of its energy queue. Moreover, due to the limited battery capacity of the WD, the sum of the available energy and the harvested energy of the WD cannot exceed the capacity of the battery, which is shown as

(23) $$\begin{array}{c} E_{m}\left(t\right)+e_{m,hw}\left(t\right)\leq \Omega ,\forall m\in M. \end{array}$$

As for the total energy consumption of the WDs in the MEC system at time slot *t*, it is defined as

(24) $$\begin{array}{c} e\left(t\right)=\sum_{m\in M}\sum_{n\in N}e_{m,total}^{n}\left(t\right). \end{array}$$

### 3.4. Optimization Problem Formulation

Based on the above model, we turn the energy minimization problem into a stochastic optimization programming problem. Especially, with the help of Lyapunov optimization theory, we turn the stochastic optimization problem P1 into a series of deterministic optimization problems, where each problem is processed in each time slot and is hoped to be solved through the standard convex optimization technology. Thus, the joint optimization of data transmission power, transmission time and need to harvest energy is made to minimize the total energy consumption of the WDs in the MEC system.

First of all, it can be learned from the previous section that the total energy consumption of the WDs in an MEC system consists of the local CPU energy consumption and the data offloaded energy consumption. Now, we denote the average time energy consumption of the WDs in an MEC system as

(25) $$\begin{array}{c} \bar{e}=\lim_{S\to +\infty }\frac{1}{S}E\left[\sum_{t=0}^{S-1}e\left(t\right)\right], \end{array}$$

where *S* is the total time length of the MEC system running.

Secondly, we model the problem of minimizing energy consumption. In order to simplify the description, we use p(t), τ(t) and e(t) to represent the set of transmission power $p_{m}\left(t\right)$, transmission time $\tau_{m}\left(t\right)$ and amount of wireless harvested energy $e_{m,w}\left(t\right)$ at time slot *t*. Moreover, we use ξ(t)=(p(t),τ(t),e(t)) to denote the set of all variables that need to be optimized at time slot *t*. Therefore, the mathematical problem of minimizing energy consumption can be modeled as follows:

(26) $$\begin{array}{c} \;\;\;\;\;\;\;\;\;\;\;P1:\underset{\;\xi (t)}{min}\bar{e},\\ s.t.(2)-(6),(12),(15),(17),(18),(22),(23). \end{array}$$

In the next section, we will give a detailed method to solve problem P1.

## 4. Online Resource Management and Allocation Optimization in MEC

In this section, in order to solve the problem of queue stability constraints P1, we use Lyapunov theory to decompose the problem into two sub-problems in a single time slot, and give the energy minimization framework. Based on this framework, an algorithm that guarantees the stability of data queues and energy queues obtains a near-optimal solution.

### 4.1. Lyapunov-Based Problem Decomposition

Within time slot *t*, the state of the MEC consists of the data queues Q(t) and energy queues E(t) of the WDs, expressed as U(t)=(Q(t),E(t)). Then, we define the Lyapunov equation L(t), which consists of the square sum of the data queue length and the remaining battery capacity:

(27) $$\begin{array}{c} L\left(t\right)=\frac{1}{2}\sum_{m\in M}\sum_{n\in N}{\left(Q_{m}^{n}\left(t\right)\right)}^{2}+\frac{1}{2}\sum_{m\in M}{\left(-{\hat{E}}_{m}\left(t\right)\right)}^{2}, \end{array}$$

where ${\hat{E}}_{m}\left(t\right)=\Omega -E_{m}\left(t\right)$ represents the remaining battery capacity of WD *m*. L(t) is a scalar measure of the length of the data queue and the size of the remaining battery capacity. It is known from Equation (27), the smaller the value of L(t), the smaller the length of the data queues, which also indicates that the remaining batteries capacity of the WDs is low, and vice versa. In addition, we define the Lyapunov drift ΔL(t), which represents the expectation of the deviation of the Lyapunov equations between time slot t+1 and time slot *t* when given the network state U(t), i.e.,

(28) $$\begin{array}{c} \Delta L(t)=E[L(t+1)-L(t)|U(t)]. \end{array}$$

In order to achieve the goal of minimizing the energy consumption of the WDs, we integrate the energy consumption function into the Lyapunov drift ΔL(t) to obtain the drift plus energy consumption function $\Delta_{V}L\left(t\right)$:

(29) $$\begin{array}{c} \Delta_{V}L\left(t\right)=\Delta L\left(t\right)+V\cdot E\left[e\left(t\right)|U\left(t\right)\right], \end{array}$$

where *V* is a non-negative weight, and it represents the proportion of energy consumption e(t) in $\Delta_{V}L\left(t\right)$. The higher the value of *V*, the higher the proportion of e(t) in $\Delta_{V}L\left(t\right)$, and vice versa. By minimizing $\Delta_{V}L\left(t\right)$, the purpose of stabilizing queue length and minimizing energy consumption can be achieved jointly. However, as the value of *V* increases, the length of data queues and energy queues also increases. In other words, WDs require larger data storage devices and batteries to ensure the running of the MEC. By adjusting the value of *V*, we can achieve a trade-off between queue length and energy consumption in the MEC system.

Since the function $\Delta_{V}L\left(t\right)$ is a quadratic function of the variables in ξ(t), which are transmission power p(t), transmission time τ(t) and amount of energy harvested e(t). Thus, it is very difficult to minimize $\Delta_{V}L\left(t\right)$. For easier optimization of these variables, Lemma 1 gives the upper limitation of $\Delta_{V}L\left(t\right)$ in time slot *t*. The upper limitation is a linear function composed of the variables to be optimized, thus it can greatly reduce the difficulty of the solution, and indirectly minimize $\Delta_{V}L\left(t\right)$.

**Lemma** **1.** *For any feasible ξ(t), the upper bound of ΔL(t) is showed as:*

(30) $$\begin{array}{cc} \Delta L(t)\leq & C^{max}-\sum_{m\in M}\sum_{n\in N}Q_{m}^{n}\left(t\right)E\left\{D_{m}^{n}\left(t\right)|U\left(t\right)\right\}+\sum_{m\in M}{\hat{E}}_{m}\left(t\right)E\left\{e_{m,hw}\left(t\right)|U\left(t\right)\right\}, \end{array}$$

*where*

(31) $$\begin{array}{cc} C^{max}= & \frac{1}{2}\sum_{m\in M}\sum_{n\in N}{\left(e_{max}\right)}^{2}+{\left(\Omega \right)}^{2}+{\left(D_{m}^{n,max}\right)}^{2}+{\left(I_{m}^{n,max}\right)}^{2})+\sum_{m\in M}\sum_{n\in N}Q_{m}^{n}\left(t\right)I_{m}^{n}\left(t\right). \end{array}$$

**Proof** **of** **Lemma** **1.** The proof is provided in Appendix A. ☐

Then, we can know the upper bound of $\Delta_{V}L\left(t\right)$ that is following as:

(32) $$\begin{array}{cc} \Delta_{V}L\left(t\right)\leq & C^{max}+E\left[Ve\left(t\right)-\sum_{m\in M}\sum_{n\in N}Q_{m}^{n}\left(t\right)D_{m}^{n}\left(t\right)+\sum_{m\in M}{\hat{E}}_{m}\left(t\right)e_{m,hw}\left(t\right)|U\left(t\right)\right]. \end{array}$$

In order to minimize the total energy consumption of WDs in the MEC system and ensure the stability of the queue at the same time, we minimize the upper bound of $\Delta_{V}L\left(t\right)$. In addition, since we consider the queue state U(t) within a single time slot *t*, we can remove expectations. In other words, we convert P1 to P2 based on Lemma 1 as follows:

(33) $$\begin{array}{cc} P2:\underset{\xi (t)}{min} & Ve\left(t\right)-\sum_{m\in M}\sum_{n\in N}Q_{m}^{n}\left(t\right)D_{m}^{n}\left(t\right)+\sum_{m\in M}{\hat{E}}_{m}\left(t\right)e_{m,hw}\left(t\right)\\ & s.t.(2)-(6),(12),(15),(17),(18),(22),(23). \end{array}$$

Problem P2 is consisting of two parts linearly. Therefore, we minimize the problem into two sub-problems which are transmission optimization and battery management. In the sub-problem of transmission optimization, we optimize the transmission power p(t) and transmission time τ(t) during tasks offloaded, while we optimize the amount of harvested energy e(t) in the sub-problem of energy management. After solving these two sub-problems, WDs update their own data queues and energy queues to prepare for the optimization of the next time slot.

We will study the solution of these two sub-problems in the following sections.

#### 4.1.1. Transmission Optimization Problem

Considering the first term and the second term in problem P2, the following transmission optimization problem can be designed to solve the transmission power p(t) and the transmission time τ(t),

(34) $$\begin{array}{cc} \underset{p(t),\tau (t)}{max} & \sum_{m\in M}\sum_{n\in N}Q_{m}^{n}\left(t\right)D_{m}^{n}\left(t\right)-Ve\left(t\right)\\ & s.t.(2)-(6),(12),(17),(18),(22). \end{array}$$

However, the maximization problem (34) is not a convex optimization problem because of the constraint (20). In order to make problem (34) easier to handle, we introduce a set of auxiliary variables $e^{\mathrm{var}}\left(t\right)$, i.e., $e_{m}^{n,var}\left(t\right)=p_{m}\left(t\right)\tau_{m}^{n}\left(t\right)$. Therefore, we convert problem (34) to problem (35), which is displayed as follows:

(35) $$\begin{array}{cc} \underset{e^{\mathrm{var}}\left(t\right),\tau \left(t\right)}{max} & \sum_{m\in M}\sum_{n\in N}\{Q_{m}^{n}\left(t\right)T\frac{f_{m}^{n}\left(t\right)}{L_{m}\left(t\right)}+Q_{m}^{n}\left(t\right)B_{m,ul}\tau_{m}^{n}\left(t\right)\log_{2}\left(1+\frac{e_{m}^{n,var}\left(t\right)h_{m,ul}\left(t\right)}{\tau_{m}^{n}\left(t\right)w_{0}}\right)\\ & -V(e_{m}^{n,var}\left(t\right)+\kappa {\left[f_{m}^{n}\left(t\right)\right]}^{3}T)\}\\ & s.t.\left\{\begin{array}{c} \left(2)-(6),(12),(17),(18\right)\\ \sum_{n\in N}\left\{e_{m}^{n,var}\left(t\right)+\kappa {\left[f_{m}^{n}\left(t\right)\right]}^{3}T\right\}\leq E_{m}\left(t\right). \end{array}\right. \end{array}$$

**Lemma** **2.** *Problem (35) is a convex optimization problem.*

**Proof** **of** **Lemma** **2.** The proof is provided in Appendix B. ☐

Due to problem (35) being a convex optimization problem, it can be solved by a standard convex optimization technique, such as the interior point method. In our paper, we use the interior point method to solve the objection function in problem (35), and the computational complexity is roughly proportional to O(max((MN+M+1),F)), where (MN+M+1) is the total size of set $e^{\mathrm{var}}\left(t\right)$ and τ(t), while *F* denotes the cost of evaluating the objection function, first and second derivatives.

#### 4.1.2. Battery Management Problem

Considering the third term in problem P2, a battery management problem can be constructed to solve $e_{m,w}\left(t\right),$ which represents the wireless energy amount, which is harvested by WD *m* at time slot *t*, respectively:

(36) $$\begin{array}{cc} \underset{e(t)}{min} & \sum_{m\in M}{\hat{E}}_{m}\left(t\right)e_{m,hw}\left(t\right),\\ & s.t.(15),(17),(18),(23). \end{array}$$

The battery management problem is a linear programming problem. In order to achieve the equation $e_{m,hw}\left(t\right)=\Omega -E_{m}\left(t\right)$ as much as possible, let $e_{m,w}^{*}\left(t\right)$ be the optimal solution to the battery management problem and the corresponding energy transfer time is defined as ${l_{m}}^{*}\left(t\right)$. If the battery of WD *m* can accommodate more energy $E_{m}\left(t\right)<\Omega$ at the beginning of time slot *t*, that is to say ${\hat{E}}_{m}\left(t\right)>0$, then we need to get the wireless energy $e_{m,w}\left(t\right)=max\left\{\Omega -E_{m}\left(t\right)-\gamma_{m}\left(t\right),0\right\}$ from the AP; otherwise, the AP will not transfer energy to WD *m*, meaning $e_{m,hw}\left(t\right)=0$.

When $E_{m}\left(t\right)<\Omega$, we can know $\ell_{m}\left(t\right)\leq T-\underset{n\in N}{\sum }\tau_{m}^{n}\left(t\right)$ from Equation (17) and make $\ell_{m}\left(t\right)=T-\underset{n\in N}{\sum }\tau_{m}^{n}\left(t\right)$. Thus, ${\ell_{m}}^{*}\left(t\right)=min\left\{\ell_{m},\frac{e_{m,w}\left(t\right)}{\mu p_{ap}\left(t\right)h_{m,dl}\left(t\right)}\right\}$ and $e_{m,w}^{*}\left(t\right)=\mu p_{ap}\left(t\right)h_{m,dl}\left(t\right){\ell_{m}}^{*}\left(t\right)$. In short, by solving the battery management problem, the WD will get as much energy as possible to fill the battery.

### 4.2. Energy Consumption Minimization Resource Management and Allocation Algorithm (ECM-RMA)

Based on the analysis of the above two parts, we propose the ECM-RMA algorithm. In addition, the process description is given in Algorithm 1. The MEC system executes the ECM-RMA algorithm to obtain the optimal wireless transmission power $p^{*}\left(t\right)$, transmission time $\tau^{*}\left(t\right)$, and amount of wireless harvested energy $e^{*}\left(t\right)$ at each time slot, respectively. Then, the length of data queue Q(t) and energy queue E(t) are updated according to the value of their respective variable.

Because the battery management problem has a closed-form solution, the complexity is negligible. In that way, transmission optimization problem determines the complexity of the ECM-RMA algorithm. Hence, the time complexity of the ECM-RMA algorithm is O(max((MN+M+1),F)).

**Algorithm 1** Proposed ECM-RMA algorithm
**Input:**
$Q\left(t\right),E\left(t\right),B_{m,ul},h_{m,ul}\left(t\right),w_{0},f_{m}^{n}\left(t\right),L_{m}\left(t\right),T,\forall m\in M,\forall n\in N$

**Output:**
$p^{*}\left(t\right),\tau^{*}\left(t\right),e^{*}\left(t\right),Q\left(t+1\right),E\left(t+1\right)$
1:/∗ Transmission Power and Transmission Time Allocation ∗/2:
**for**
m∈M
**do**
3:     **for**
n∈N
**do**  4:         Solve the standard convex optimization problem in (35) to acquire the optimal power and time allocation policy;  5:     **end for**6:**end for** 7:/∗ Battery Management ∗/ 8:**for**m∈M**do**  9:     **if**
$E_{m}\left(t\right)<\Omega$
**then**
10:         $e_{m,w}\left(t\right)=max\left\{\Omega -E_{m}\left(t\right)-\gamma_{m}\left(t\right),0\right\}$; 11:         $\ell_{m}\left(t\right)=T-\underset{n\in N}{\sum }\tau_{m}^{n}\left(t\right)$; 12:         ${\ell_{m}}^{*}\left(t\right)=min\left\{\ell_{m},\frac{e_{m,w}\left(t\right)}{\mu p_{ap}\left(t\right)h_{m,dl}\left(t\right)}\right\}$; 13:         $e_{m,w}^{*}\left(t\right)=\mu p_{ap}\left(t\right)h_{m,dl}\left(t\right){\ell_{m}}^{*}\left(t\right)$; 14:     **else** 15:         $e_{m,w}^{*}\left(t\right)=0$
16:     **end if** 17:**end for** 18:/∗ Update the Queue Length ∗/19:
**for**
m∈M
**do**
20:     Compute $E_{m}\left(t+1\right)$ based on Equation (11);  21:     **for**
n∈N
**do**
22:         Compute $Q_{m}^{n}\left(t+1\right)$ based on Equation (14);  23:     **end for** 24:
**end for**


### 4.3. Performance Analysis

We analyze the performance of the ECM-RMA algorithm in this section. As mentioned in the previous parts, the upper limitation of $\Delta_{V}L\left(t\right)$ is indirectly minimizing $\Delta_{V}L\left(t\right)$. The proposed ECM-RMA algorithm is used to solve the problem P2 in (31), which is not equal to original problem P1. Thus, how is the performance of the proposed ECM-RMA algorithm? We first show the performance of the proposed ECM-RMA algorithm in Theorem 1. Then, we reveal the energy consumption and delay trade-off achieved by the proposed ECM-RMA algorithm in Theorem 2.

#### 4.3.1. The Performance of the Proposed ECM-RMA Algorithm

**Lemma** **3.** *We assume that problem P1 is reasonable, i.e., given average arrival data rate $\lambda_{m}^{n}$, there is at least one transmission power and time allocation scheme that satisfies all the limitations in P1. For a given average data arrival rate $\lambda_{m}^{n}+\varepsilon$, where ε is a positive number and P1 is also feasible. Then, for any ς>0, there is at least one power and time allocation scheme $\Theta^{alg.1}=\left(p_{m}\left(t\right),\tau_{m}^{n}\left(t\right)\right)$ that satisfies the following restrictions:*

(37) $$\begin{array}{c} E\left(e^{alg.1}\left(t\right)|U\left(t\right)\right)={\bar{e}}^{alg.1}\leq {\bar{e}}^{opt}+\varsigma , \end{array}$$

(38) $$\begin{array}{c} E\left(D^{alg.1}\left(t\right)|U\left(t\right)\right)=E\left({\bar{D}}^{alg.1}\left(t\right)\right)\geq \lambda_{m}^{n}+\varepsilon , \end{array}$$

*where ${\bar{e}}^{alg.1}$ and ${\bar{e}}^{opt}$ represent the average time energy consumption under ECM-RMA algorithm (Algorithm 1) and the optimal value for solving the original problem P1, respectively. $e^{alg.1}\left(t\right)$ and $D^{alg.1}\left(t\right)$ represent the energy consumption and the amount of data processed in time slot t under the scheme $\Theta^{alg.1}$, respectively.*

**Proof** **of** **Lemma** **3.** For brevity, the proof process is omitted and similar proof in [32,33,34]. ☐

**Assumption** **1.** *Next, we assume that there is an upper and lower bound on the average time energy consumption by ECM-RMA algorithm, i.e., ${\bar{e}}^{min}\leq {\bar{e}}^{alg.1}\leq {\bar{e}}^{max}$. Similar assumptions can be found in [32,33,34].*

Using Lemma 3, we can get the gap between $\;{\bar{e}}^{alg.1}$ and ${\bar{e}}^{opt}$, as shown in the following Theorem 1.

**Theorem** **1.** *When P1 is reasonable and V>0, the boundary of ${\bar{e}}^{alg.1}$ is as follows:*

(39) $$\begin{array}{c} {\bar{e}}^{opt}\leq {\bar{e}}^{alg.1}\leq {\bar{e}}^{opt}+\frac{c^{max}}{V}, \end{array}$$

*where $c^{max}=\frac{1}{2}\sum_{m\in M}\sum_{n\in N}\left({\left(e_{max}\right)}^{2}+{\left(\Omega \right)}^{2}+{\left(D_{m}^{n,max}\left(t\right)\right)}^{2}+{\left(I_{m}^{n,max}\left(t\right)\right)}^{2}\right)$ derived from the Proof of Lemma 1 in Appendix A.*

**Proof** **of** **Theorem** **1.** The proof is provided in Appendix C. ☐

Theorem 1 suggests the upper and lower bounds of the average time energy consumption ${\bar{e}}^{alg.1}$ is obtained by the proposed ECM-RMA algorithm. Specifically, the time average energy consumption ${\bar{e}}^{alg.1}$ decreases at the speed of O(1/V). Thus, by setting a sufficiently large *V* value, the average time energy consumption ${\bar{e}}^{alg.1}$ obtained by the ECM-RMA algorithm can be arbitrarily close to the optimal value of the original problem P1.

#### 4.3.2. The Trade-Off between Energy Consumption and Delay

In order to reveal the energy consumption and delay trade-off achieved by the proposed ECM-RMA algorithm, we will describe the performance based on the time average data queue length, which is given in the following Theorem 2.

**Theorem** **2.** *We assume that problem P1 is reasonable, and then, in the considered MEC system, the ECM-RMA algorithm ensures that the all data queues are stable. The upper bound of the average time data queue length is shown as follows:*

(40) $$\begin{array}{cc} \bar{Q}= & \lim_{S\to +\infty }\frac{1}{S}\sum_{t=0}^{S-1}\sum_{m\in M}\sum_{n\in N}Q_{m}^{n}\left(t\right)\leq \frac{c^{max}+V\left({\bar{e}}^{opt}-{\bar{e}}^{min}\right)}{\varepsilon }, \end{array}$$

*where ${\bar{e}}^{min}$ is the lower bound of ${\bar{e}}^{opt}$, the assumption made in Section 4.3.1.*

**Proof** **of** **Theorem** **2.** The proof is provided in Appendix D. ☐

The average time power consumption ${\bar{e}}^{alg.1}$ drops at a rate of O(1/V) seen in Theorem 1, and, as seen from Theorem 2, the average data queue length increases at a rate of O(V). In other words, there is a trade-off between the average time energy consumption and the average time data queue length. In detail, with a large *V* value, there is less time average energy consumption but a larger average time data queue length. In addition, the trade-off can be described quantitatively as [O(1/V),O(V)]. In addition, $\bar{T}$ is denoting the average time queue delay, which is proportional to the average time data queue length at a given data arrival rate, i.e., $\bar{T}=\lim_{S\to +\infty }\frac{1}{S}\sum_{t=0}^{S-1}\sum_{m\in M}\sum_{n\in N}\frac{Q_{m}^{n}\left(t\right)}{\lambda_{m}^{n}}$. Hence, the proposed ECM-RMA algorithm achieves an energy consumption-delay trade-off as [O(1/V),O(V)] too. Obviously, the system resources can be effectively used, and the service quality of the user can be guaranteed by controlling the system parameter *V*.

## 5. Simulation Results

In this section, we use Matlab software (R2016a, MathWorks, Natick, Massachusetts, MA, USA) for simulation and the results of simulation are provided to evaluate the performance of the proposed ECM-RMA algorithm. We verify the reasonability and high efficiency of the ECM-RMA algorithm in minimizing energy consumption.

### 5.1. Simulation Settings in All Simulations, the Energy Transmitter at the AP with $p_{ap}=3$ W

We consider a Small-scale Rayleigh fading channel model, and the channel gain $h_{i}={\bar{h}}_{i}\alpha$. Here, ${\bar{h}}_{i}$ indicates that the average channel gain is determined by the geographical position of WD *m* and α is a random variable of a unit mean independent exponential. Specifically, ${\bar{h}}_{i}$ follows the model below:

(41) $$\begin{array}{c} {\bar{h}}_{m,dl}=A_{d}\left(\frac{3\cdot 10^{8}}{4\pi f_{c}d_{m}}\right){}^{d_{e}},\forall m\in M. \end{array}$$

This is a free-space path loss model, where $A_{d}=4.11$ indicates the antenna gain, $f_{c}=915$ MHz indicates the carrier frequency. The unit of $d_{e}$ is meters, which means the distance between WD *m* and the AP. In addition, $d_{e}\geq 2$ indicates the path loss exponent. Here, we set $d_{e}=2.8$. For ease of explanation, we consider $h_{m,ul}=h_{m,dl}$, $d_{m}=2.5+0.3\left(m-1\right)$ meters and static channel model with α=1. In this way, $h_{m,ul}=h_{m,dl}={\bar{h}}_{m,dl}$ and channel gain decreases with the increasing *m*.

As for some parameters of the energy harvesting from the environment, we set the maximum green energy supply $\gamma_{max}=2$, and the green energy supply $\gamma_{m}$ is uniformly distributed in $\left[0,\gamma_{max}\right]$. Each WD has the same parameters, Ω=10 J, $k=10^{-18}$, $f_{m}^{n}=2.4$ GHz and $L_{m}=788$ cycles/bit. In addition, each WD has the same energy queue size $R^{E}=\Omega =10$ J, and each WD allocates the same amount of data storage space $R^{D}=1000$ bits/Hz for each task. $R^{D}=1000$ bits/Hz is the available data queue length of each task. Moreover, the completion deadline required by each WD is the same, $J_{m}=0.78$ s. In addition, each task requires the same computing resources, $g_{m}^{n}=0.5$ GHz. As for the computation on the MEC server, the computation capability of the MEC server is $f_{ap}=4$ GHz/s [35]. During the communication process, $B_{m,ul}=1$ MHz, $w_{0}=10^{-10}$ W, $p_{m,min}=0.1$ Wh and $p_{m,max}=0.2$ Wh.

The length of each time slot T=1 s, and the total time length of all simulations S=10,000 s. At the beginning of all simulations, we set $Q_{m}^{n}\left(0\right)=0$ and $E_{m}\left(0\right)=R^{E},\forall m\in M,\forall n\in N$. Without loss of generality, the data arrival for all tasks are set to a random process with the equal average data arrival rates, i.e., $\lambda_{m}^{n}=\lambda$. Unless otherwise stated, λ=120 bits/time-slot, K=2, μ=0.5.

To evaluate the efficiency of the proposed ECM-RMA algorithm, we compare the ECM-RMA algorithm with the other two strategies, which are Baseline 1 and Baseline 2. Baseline 1 assigns equal time to tasks, and only optimizes transmission power allocation of tasks. As for Baseline 2, it only considers that WDs harvest energy from the environment. Unless otherwise stated, the other optimizations of Baseline 1 and Baseline 2 are consistent with the ECM-RMA algorithm.

### 5.2. Performance Achieved by the ECM-RMA Algorithm

**Figure 3:**
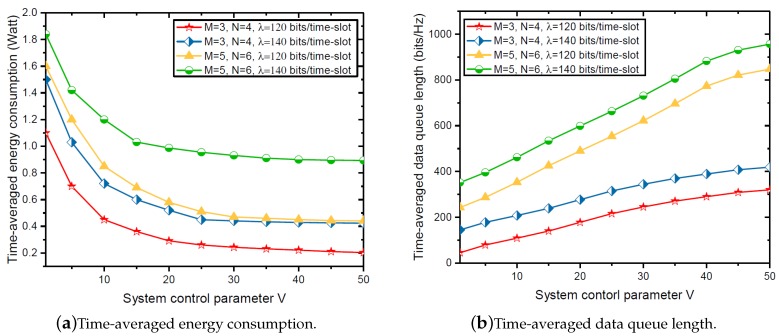
Time-averaged energy consumption and data queue length versus system control parameter *V*.

Figure 3a shows the time-averaged energy consumption versus the system control parameter *V* ranging from 1 to 50. It can be seen from Figure 3 that the time-averaged energy consumption decreases with the speed of O(1/V) as *V* increases, which confirms the viewpoint in Theorem 1. In particular, when *V* is large enough, the time-averaged energy consumption eventually converges to the optimal value of problem P1, which indicates that the proposed ECM-RMA algorithm is asymptotically optimal for solving problem P1. In addition, we can observe that, as the number of WDs *M* and the number of tasks *N* increase, the time-averaged energy consumption will also increase. This is due to the fact that, during a certain length of time, a larger number of WDs *M* and a larger number of tasks *N* require a larger transmission rate to ensure the stability of the task data queue, which results in much more time-averaged energy consumption. In addition, part of the time-averaged energy consumption (10) can explain that the above analysis is reasonable.

Figure 3b shows the time-averaged task data queue length versus the system control parameter *V* ranging from 1 to 50. Expectedly, the time-averaged data queue length increases with the speed of O(V) as *V* increases, which confirms the viewpoint in Theorem 2. Furthermore, we can observe that the time-averaged task data queue increases as the number of WDs *M*, the number of tasks *N* and the average data arrival rate λ increase. This is because more WDs, tasks and larger average data arrival rate λ mean more data that need to be processed, which results in an increase in the length of the data queue.

**Figure 4:**
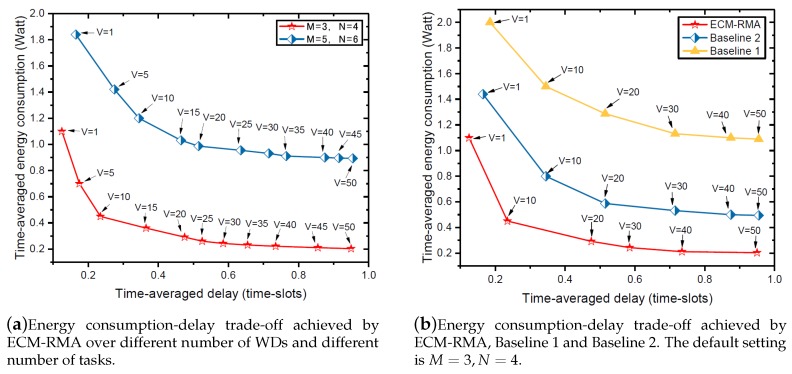
Energy consumption-delay trade-off.

**Figure 5:**
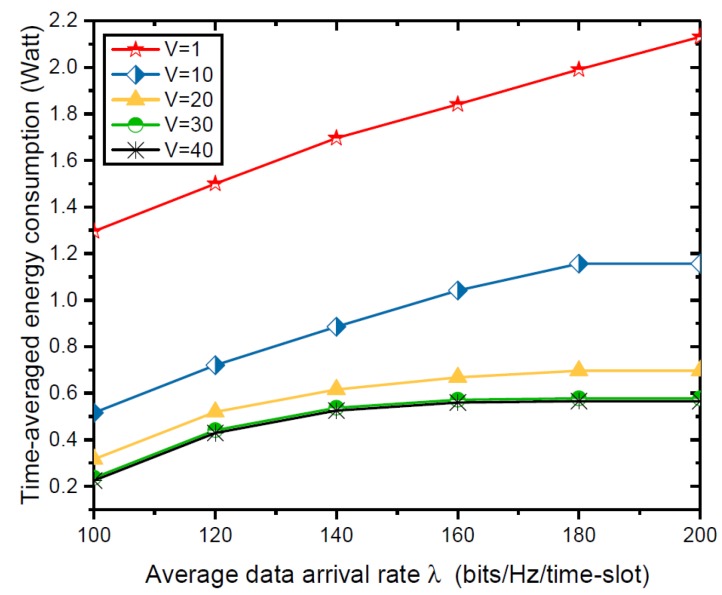
Time-averaged energy consumption versus average data arrival rate λ.

Figure 4a shows the energy consumption-delay trade-off for the WDs in the MEC system. Obviously, the larger the time-averaged delay, the smaller the time-averaged energy consumption. This is because a larger time-averaged delay means a lower quality of service (QoS), which requires only a smaller transmission rate $\nu_{m}\left(t\right)$. According to (1) and (10), only a small transmission power is required, which results in lesser energy consumption. Based on the analysis in Figure 3, Figure 4 and Figure 5, there is a trade-off between energy consumption and delay, and it is quantized as [O(1/V),O(V)]. By adjusting the value of *V*, we can effectively control the performance of energy consumption-delay in the MEC system. More specifically, if the devices in the system have a high requirement for low latency, then a smaller *V* value needs to be set. Conversely, if the devices have a high requirement for low energy consumption, a large *V* value needs to be set.

Figure 4b shows the energy consumption-delay trade-off achieved by ECM-RMA, Baseline 1 and Baseline 2. As expected, the proposed ECM-RMA algorithm is better than Baseline 1 and Baseline 2. In more detail, in the case of the same delay, the proposed ECM-RMA algorithm consumes less energy than Baseline 1 and Baseline 2, and this also means that the ECM-RMA algorithm has the lowest latency when the same energy is consumed. This is because, compared to Baseline 1, the ECM-RMA algorithm can allocate transmission time according to the dynamics of channel and the length of date queue. Under Baseline 2, the energy harvested by WDs is unstable, and WDs cannot compute tasks, instantly resulting in long delays when the same energy is consumed.

### 5.3. Impact of λ, K, μ

Now, we evaluate the impact of system variables λ, *K*, μ on the time-averaged energy consumption. Unless otherwise stated, M=3, N=4. Figure 5 shows the time-averaged energy consumption versus the average data arrival rate λ ranging from 100 to 200 under different system control parameter *V*. The time-averaged energy consumption increases with increasing λ. Meanwhile, the rate of increase in the time-averaged energy consumption slows down when there is a higher average data arrival rate λ due to the limited data queue size and the limited available battery capacity.

**Figure 6:**
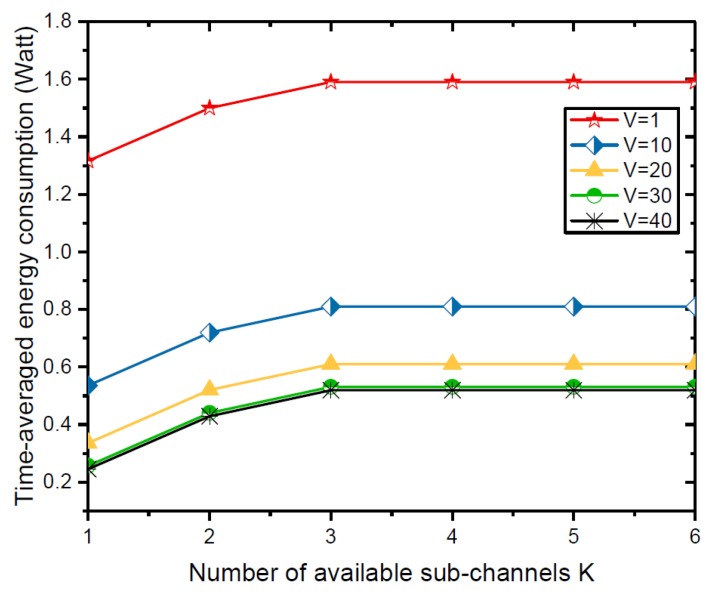
Time-averaged energy consumption versus number of available sub-channels *K*.

Figure 6 shows the time-averaged energy consumption versus the number of available sub-channels *K* under different system control parameter *V*. Since more available sub-channels can support more WDs for data transmission, the time-averaged energy consumption increases with *K* when K≤M, i.e., the number of available sub-channels does not exceed the number of WDs in the MEC system.

**Figure 7:**
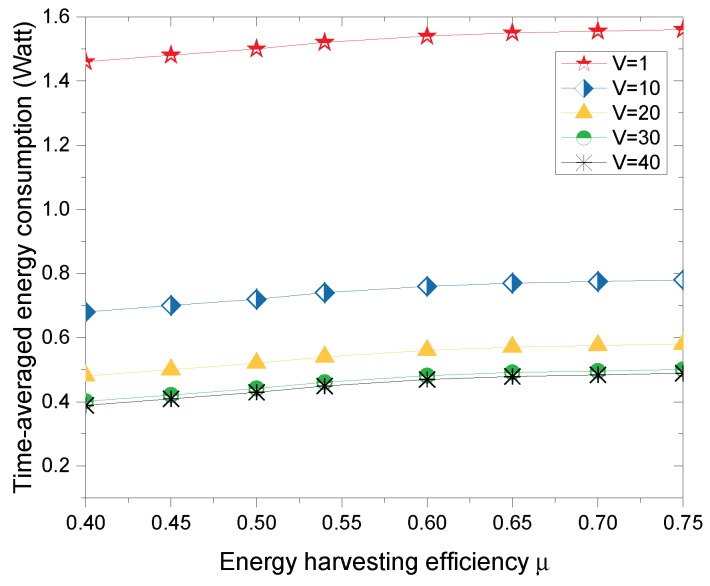
Time-averaged energy consumption versus energy harvesting efficiency μ.

Figure 7 shows the time-averaged energy consumption versus the energy harvesting efficiency μ under different system control parameter *V*. The time-averaged energy consumption monotonically increases with the increase of energy harvesting efficiency μ. This is because the wireless harvested energy of WDs is a linear function positively correlated with the energy harvesting efficiency μ according to (16), and, as μ increases, more energy can be used for wireless data transmission. However, the growth rate of the time-average energy consumption slows down with higher μ. This indicates that the time-averaged energy consumption is also affected by the battery capacity and the number of available sub-channels in case of sufficient energy supply.

## 6. Conclusions

In this paper, we investigated the time-averaged energy consumption minimization problem in a multi-user multi-task MEC system with hybrid energy harvest IoT devices. A time-average energy consumption minimization problem while satisfying data queue stability and harvesting energy availability constraints was formulated as an online stochastic optimization problem. We propose the ECM-RMA algorithm based on Lyapunov optimization technology and turn the stochastic optimization problem into two deterministic sub-problems that can be easily solved by a convex optimization technique and linear programming technique. Performance analysis shows the asymptotic optimization of the proposed ECM-RMA algorithm and energy consumption and delay trade-off. The simulation results verify the theoretical analysis and the effectiveness of the proposed ECM-RMA algorithm in minimizing the time-averaged energy consumption.

## Figures and Tables

**Table 1:** Definition of notations.

Notation	Defination
M	The set of WDs
N	The set of tasks
T	The set of time slots
$p_{m}\left(t\right)$	The uplink transmission power between WD *m* and the AP at time slot *t*
$h_{m,ul}\left(t\right)$	The uplink channel gains between WD *m* and the AP at time slot *t*
$w_{0}$	The white noise power level of uplink channel
$B_{m,ul}$	The uplink channel bandwidth that the AP allocates for the WD *m* at time slot *t*
$\nu_{m}\left(t\right)$	The uplink transmission rate between WD *m* and the AP
$\tau_{m}^{n}\left(t\right)$	The duration of task *n* partially offloaded from WD *m* to the AP within time slot *t*
$\tau_{m}\left(t\right)$	The time allocated schedule of all tasks in WD *m* during slot *t*
$I_{m}^{n}\left(t\right)$	The amount of computation task arrived at user *m* at the end of the time slot *t*
$f_{m}^{n}\left(t\right)$	The CPU-cycle frequency assigned to task *n* on WD *m* at time slot *t*
$L_{m}\left(t\right)$	The CPU-cycles required to calculate one bit of data in WD *m* at time slot *t*
$D_{m,l}^{n}\left(t\right)$	The local computed data amount of task *n* of WD *m* at time slot *t*
$e_{m,l}^{n}\left(t\right)$	The energy consumption for local computation for task *n* of WD *m* during a time slot *t*
$D_{m,c}^{n}\left(t\right)$	The offloaded data amount of task *n* of WD *m* at time slot *t*
$e_{m,c}^{n}\left(t\right)$	The energy consumption of WD *m* offloading the data of task *n* at time slot *t*
$D_{m}^{n}\left(t\right)$	The total computed data of task n of WD *m* at time slot *t*
$Q_{m}^{n}\left(t\right)$	The backlog of the data queue for task *n* of WD *m* at time slot *t*
$e_{m,h}\left(t\right)$	The energy obtained from the environment of WD *m* at time slot *t*
$e_{m,w}\left(t\right)$	The energy obtained from the AP of WD *m* at time slot *t*
$e_{m,hw}\left(t\right)$	The total harvested energy of WD *m* at time slot *t*
γ(t)	The energy supply from the environment at time slot *t*
$e_{m,total}^{n}\left(t\right)$	The total energy consumed by WD *m* for task n within time slot *t*
$E_{m}\left(t\right)$	The dynamic change of WD’s energy queue
*M*	The number of WDs
*N*	The number of tasks in a WD
*T*	The slot length
*S*	The number of slots
*K*	The number of available sub-channel
$p_{m,max}$	The maximum uplink transmission power
$p_{m,min}$	The minimum uplink transmission power
$I_{m}^{n,max}$	The maximum arrive data
$\gamma_{max}$	The maximum green energy supply

**Table 2:** Definition of abbreviations used in the Table 1.

Abbreviation	Defination
WD	Wireless device
WDs	Wireless devices
AP	Access point

## Abbreviations

The following abbreviations are used in this manuscript:

MEC	mobile edge computing
WDs	wireless devices
IoT	Internet of Things
ECM-RMA	energy-efficient joint resource management and allocation
AP	access point
EH	energy harvesting
RF	radio frequency
WPT	wireless power transfer
MDP	Markov decision processes
eDors	energy-efficient dynamic offloading resource scheduling
i.i.d	independent and identically distributed
QoS	quality of service

## Appendix A. Proof of Lemma 1

Because ${\left(max[a-b,0]+c\right)}^{2}\leq a^{2}+b^{2}+c^{2}-2a\left(b-c\right)$, we square both sides of (15) and (21) to get (A1) and (A2), respectively. In addition, (A1) and (A2) are shown below:

(A1) $$\begin{array}{cc} Q_{m}^{n}{\left(t+1\right))}^{2}\leq & {\left(Q_{m}^{n}\left(t\right)\right)}^{2}+{\left(D_{m}^{n}\left(t\right)\right)}^{2}+{\left(I_{m}^{n}\left(t\right)\right)}^{2}-2Q_{m}^{n}\left(t\right)\left(D_{m}^{n}\left(t\right)-I_{m}^{n}\left(t\right)\right), \end{array}$$

(A2) $$\begin{array}{cc} {\left({\hat{E}}_{m}\left(t+1\right)\right)}^{2}\leq & {\left({\hat{E}}_{m}\left(t\right)\right)}^{2}+{\left(\sum_{n\in N}e_{m,total}^{n}\left(t\right)\right)}^{2}+{\left(e_{m,hw}\left(t\right)\right)}^{2}-2{\hat{E}}_{m}\left(t\right)\left(\sum_{n\in N}e_{m,total}^{n}\left(t\right)-e_{m,hw}\left(t\right)\right). \end{array}$$

Summing all WDs m∈{1,2,…M} for (A1) and (A2), we can get (A3) and (A4), respectively. In addition, (A3) and (A4) are shown below:

(A3) $$\begin{array}{c} \sum_{m\in M}\sum_{n\in N}\frac{1}{2}\left({\left(Q_{m}^{n}\left(t+1\right)\right)}^{2}-{\left(Q_{m}^{n}\left(t\right)\right)}^{2}\right)\leq \\ \frac{1}{2}\sum_{m\in M}\sum_{n\in N}\left({\left(D_{m}^{n}\left(t\right)\right)}^{2}+{\left(I_{m}^{n}\left(t\right)\right)}^{2}\right)-\sum_{m\in M}\sum_{n\in N}Q_{m}^{n}\left(t\right)\left(D_{m}^{n}\left(t\right)-I_{m}^{n}\left(t\right)\right), \end{array}$$

(A4) $$\begin{array}{c} \sum_{m\in M}\frac{1}{2}{\left({\left({\hat{E}}_{m}\left(t+1\right)\right)}^{2}-{\hat{E}}_{m}\left(t\right)\right)}^{2})\leq \\ \frac{1}{2}\sum_{m\in M}\left({\left(\sum_{n\in N}e_{m,total}^{n}\left(t\right)\right)}^{2}+{\left(e_{m,hw}\left(t\right)\right)}^{2}\right)-\sum_{m\in M}{\hat{E}}_{m}\left(t\right)\left(\sum_{n\in N}e_{m,total}^{n}\left(t\right)-e_{m,hw}\left(t\right)\right). \end{array}$$

Therefore, the Lyapunov drift ΔL(t) can be given by

(A5) $$\begin{array}{c} \Delta L(t)\leq \\ \frac{1}{2}\sum_{m\in M}\sum_{n\in N}E\left\{\left({\left(D_{m}^{n}\left(t\right)\right)}^{2}+{\left(I_{m}^{n}\left(t\right)\right)}^{2}\right)|U\left(t\right)\right\}-\sum_{m\in M}\sum_{n\in N}Q_{m}^{n}\left(t\right)E\left\{\left(D_{m}^{n}\left(t\right)-I_{m}^{n}\left(t\right)\right)|U\left(t\right)\right\}\\ +\frac{1}{2}\sum_{m\in M}E\left\{\left({\left(\sum_{n\in N}e_{m,total}^{n}\left(t\right)\right)}^{2}+{\left(e_{m,hw}\left(t\right)\right)}^{2}\right)|U\left(t\right)\right\}-\sum_{m\in M}{\hat{E}}_{m}\left(t\right)E\left\{\left(\sum_{n\in N}e_{m,total}^{n}\left(t\right)-e_{m,hw}\left(t\right)\right)|U\left(t\right)\right\}. \end{array}$$

During a time slot, a fixed amount of data can be processed in a WD according to Equation (7). In addition, the amount of data to be transmitted also has upper and lower bounds. This is because the transmission power has upper and lower limits under the determined transmission time. Therefore, we know $D_{m}^{n,min}\leq D_{m}^{n}\left(t\right)\leq D_{m}^{n,max}$. In addition, $\sum_{n\in N}e_{m,total}^{n}\left(t\right)$ cannot exceed the length of its energy queue at time slot *t*, so we have the upper bound of energy consumption, e.g., $e_{max}$. As for $e_{m,hw}\left(t\right)$, it cannot exceed the size of battery capacity, so we have the upper bound of amount of harvested energy, e.g., $e_{m,hw}\left(t\right)\leq \Omega$. We use the upper bound $D_{m}^{n}\left(t\right)\leq D_{m}^{n,max},I_{m}^{n}\left(t\right)\leq I_{m}^{n,max},e_{m,total}^{n}\left(t\right)\leq e_{max},e_{m,hw}\left(t\right)\leq \gamma_{max},\forall m\in M,\forall n\in N$ and define $c^{max}=\frac{1}{2}\sum_{m\in M}\sum_{n\in N}\left({\left(e_{max}\right)}^{2}+{\left(\Omega \right)}^{2}+{\left(D_{m}^{n,max}\left(t\right)\right)}^{2}+{\left(I_{m}^{n,max}\left(t\right)\right)}^{2}\right)$. In addition, $I_{m}^{n}\left(t\right)$ is the amount of data arrive to WD *m* at the end of time slot *t*, so both the data queue length $Q_{m}^{n}\left(t\right)$ and the corresponding data queue state U(t) are independent of $I_{m}^{n}\left(t\right)$ at the current time slot *t*. As a result, $I_{m}^{n}\left(t\right)$ is independent of the current power allocation $p_{m}\left(t\right)$ and time allocation $\tau_{m}^{n}\left(t\right)$. In other words, $E\{I_{m}^{n}\left(t\right)|U\left(t\right)\}$ is a constant. We can define $C_{max}$ as follows:

(A6) $$\begin{array}{cc} C^{max}= & c^{max}+\sum_{m\in M}\sum_{n\in N}Q_{m}^{n}\left(t\right)I_{m}^{n}\left(t\right). \end{array}$$

Therefore, we have

(A7) $$\begin{array}{cc} \Delta L(t)\leq & C^{max}-\sum_{m\in M}\sum_{n\in N}Q_{m}^{n}\left(t\right)E\left\{D_{m}^{n}\left(t\right)|U\left(t\right)\right\}+\sum_{m\in M}{\hat{E}}_{m}\left(t\right)E\left\{e_{m,hw}\left(t\right)|U\left(t\right)\right\}. \end{array}$$

Lemma 1 is eventually proved.

## Appendix B. Proof of Lemma 2

First of all, we will show that the binary function $f\left(x_{1},x_{2}\right)=-x_{1}\ln\left(1+\frac{x_{2}}{x_{1}}\right)$ is convex on $x_{1}$ and $x_{2}$. The reason is as follows:

The Hessian matrix of $f\left(x_{1},x_{2}\right)=-x_{1}\ln\left(1+\frac{x_{2}}{x_{1}}\right)$ is

(A8) $$H=\left[\begin{array}{cc} \frac{x_{2}^{2}}{{\left(x_{1}+x_{2}\right)}^{2}x_{1}} & -\frac{x_{2}}{{\left(x_{1}+x_{2}\right)}^{2}}\\ -\frac{x_{2}}{{\left(x_{1}+x_{2}\right)}^{2}} & \frac{x_{1}}{{\left(x_{1}+x_{2}\right)}^{2}} \end{array}\right].$$

The eigenvalues of *H* are $k_{1}=0$ and $k_{2}=\frac{x_{1}^{2}+x_{2}^{2}}{x_{1}^{3}+2x_{1}^{2}x_{2}+x_{1}x_{2}^{2}}$, respectively, and *H* is positive semidefininite. Therefore, $f(x_{1},x_{2})$ is convex on $x_{1}$ and $x_{2}$.

For (35), let $x_{1}=\tau_{m}^{n}\left(t\right)$, $x_{2}=\frac{e_{m}^{n,var}\left(t\right)h_{m,ul}\left(t\right)}{w_{0}}$. According to (A8), since $f(x_{1},x_{2})$ is convex, $\eta_{m}^{n}\left(\tau_{m}^{n}\left(t\right),e_{m}^{n,var}\left(t\right)\right)=-\frac{Q_{m}^{n}\left(t\right)B_{m,ul}f\left(x_{1},x_{2}\right)}{\ln2}$ is concave. In the issue, $\sum_{m\in M}\sum_{n\in N}\eta_{m}^{n}\left(\tau_{m}^{n}\left(t\right),e_{m}^{n,var}\left(t\right)\right)$ is concave too. In addition, the first term in (35) is independent of the variable to be optimized, whereas the third term and fourth term are linear functions of $e_{m}^{n,var}\left(t\right)$. Therefore, the objection problem in (35) is concave. Clearly, the constraints in Equation (35) are linear of $\tau_{m}^{n}\left(t\right)$ and $e_{m}^{n,var}\left(t\right)$. Thus, the feasible region of the problem in Equation (35) is convex.

In summary, the objection problem in (35) is a convex optimization problem. Lemma 2 is eventually proved.

## Appendix C. Proof of Theorem 1

Now, we will show the performance gap caused by Lyapunov optimization theory compared to the original problem P1. First, we use Algorithm 1 to minimize the right hand of (32), and we have

(A9) $$\begin{array}{cc} \Delta_{V}L\left(t\right)\leq & C^{max}+E\left[Ve^{alg.1}\left(t\right)-\sum_{m\in M}\sum_{n\in N}Q_{m}^{n}\left(t\right)D^{alg.1}\left(t\right)+\sum_{m\in M}{\hat{E}}_{m}\left(t\right)e_{m,hw}\left(t\right)|U\left(t\right)\right]. \end{array}$$

Then, substituting (37) and (38) into (A9),

(A10) $$\begin{array}{c} \Delta_{V}L\left(t\right)\leq \\ c^{max}+\sum_{m\in M}\sum_{n\in N}Q_{m}^{n}\left(t\right)I_{m}^{n}\left(t\right)+E\left[V\left({\bar{e}}^{opt}+\varsigma \right)-\sum_{m\in M}\sum_{n\in N}Q_{m}^{n}\left(t\right)\left(\lambda_{m}^{n}+\varepsilon \right)+\sum_{m\in M}{\hat{E}}_{m}\left(t\right)e_{m,hw}\left(t\right)|U\left(t\right)\right]. \end{array}$$

In addition, because arrival data rate $\lambda_{m}^{n}$ represents the amount of data entering per second, $I_{m}^{n}\left(t\right)=\lambda_{m}^{n}$. Now, we can get

(A11) $$\begin{array}{c} \Delta_{V}L\left(t\right)\leq \\ c^{max}+E\left[V\left({\bar{e}}^{opt}+\varsigma \right)-\varepsilon \sum_{m\in M}\sum_{n\in N}Q_{m}^{n}\left(t\right)+\sum_{m\in M}{\hat{E}}_{m}\left(t\right)e_{m,hw}\left(t\right)|U\left(t\right)\right]. \end{array}$$

Setting a limit as ς→0,

(A12) $$\begin{array}{cc} \Delta_{V}L\left(t\right)\leq & c^{max}+V{\bar{e}}^{opt}-\varepsilon \sum_{m\in M}\sum_{n\in N}Q_{m}^{n}\left(t\right)+\sum_{m\in M}{\hat{E}}_{m}\left(t\right)e_{m,hw}\left(t\right). \end{array}$$

Then, accumulating (A12) from t=1 to t=S, we can get

(A13) $$\begin{array}{c} E\left[L\left(T\right)\right]-E\left[L\left(1\right)\right]+V\sum_{t\in T}E\left[e^{alg.1}\left(t\right)|U\left(t\right)\right]\leq \\ S\left(c^{max}+V{\bar{e}}^{opt}\right)-\varepsilon \sum_{t\in T}\sum_{m\in M}\sum_{n\in N}Q_{m}^{n}\left(t\right)+\sum_{t\in T}\sum_{m\in M}{\hat{E}}_{m}\left(t\right)e_{m,hw}\left(t\right). \end{array}$$

Finally, letting (A13) divide by VS and making S→∞, we can get

(A14) $$\begin{array}{c} e^{alg.1}\left(t\right)\leq {\bar{e}}^{opt}+\frac{c^{max}}{V}. \end{array}$$

Theorem 1 is eventually proved.

## Appendix D. Proof of Theorem 2

Since $Q_{m}^{n}\left(t\right)\geq 0,\forall m\in M,\forall n\in N,\forall t\in T$, we remove the second item on the right side of (A12); then, (A12) can be simplified to

(A15) $$\begin{array}{cc} \Delta_{V}L\left(t\right)\leq & c^{max}+V{\bar{e}}^{opt}+\sum_{m\in M}{\hat{E}}_{m}\left(t\right)e_{m,hw}\left(t\right), \end{array}$$

(A16) $$\begin{array}{cc} \Delta L(t)\leq & c^{max}+\sum_{m\in M}{\hat{E}}_{m}\left(t\right)e_{m,hw}\left(t\right). \end{array}$$

We learn $C_{m}^{n}\left(t\right)\leq C_{m}^{n,max}\left(t\right),\forall m\in M,\forall n\in N,\forall t\in T$, and $D_{m}^{n}\left(t\right)\leq D_{m}^{n,max},\forall m\in M,\forall n\in N,\forall t\in T$ from this paper. Thus, ΔL(t)≤A, where *A* is a finite positive constant. According to (28), we can get

(A17) $$\begin{array}{c} E[L(t+1)-L(t)|U(t)]\leq A. \end{array}$$

Accumulating (A16) from t=1 to t=T, we acquire

(A18) $$\begin{array}{c} E[L(T)-L(1)|U(t)]\leq TA. \end{array}$$

Substituting (27) into (A18), we have

(A19) $$\begin{array}{c} \frac{1}{2}E\left[\sum_{m\in M}\sum_{n\in N}{\left(Q_{m}^{n}\left(t\right)\right)}^{2}\right]+\frac{1}{2}E\left[\sum_{m\in M}{\left(-{\hat{E}}_{m}\left(t\right)\right)}^{2}\right]-E\left[L\left(1\right)\right]\leq TA, \end{array}$$

(A20) $$\begin{array}{c} E\left[\sum_{m\in M}\sum_{n\in N}{\left(Q_{m}^{n}\left(t\right)\right)}^{2}\right]\leq 2TA+2E\left[L\left(1\right)\right]-E\left[\sum_{m\in M}{\left(-{\hat{E}}_{m}\left(t\right)\right)}^{2}\right], \end{array}$$

(A21) $$\begin{array}{c} E\left[\sum_{m\in M}\sum_{n\in N}{\left(Q_{m}^{n}\left(t\right)\right)}^{2}\right]\leq 2TA+2E\left[L\left(1\right)\right]. \end{array}$$

Then, we convert (A21) to (A22)

(A22) $$\begin{array}{c} E\left[\sum_{m\in M}\sum_{n\in N}\left(Q_{m}^{n}\left(t\right)\right)\right]\leq \sqrt{2TA+2E[L(1)].} \end{array}$$

Letting (A22) divide by *S* and making S→∞, we can get

(A23) $$\begin{array}{c} \lim_{S\to +\infty }\frac{1}{S}E\left[\sum_{m\in M}\sum_{n\in N}\left(Q_{m}^{n}\left(t\right)\right)\right]<\infty . \end{array}$$

From (A2), we know that the ECM-RMA algorithm can guarantee the stability of all data queues in the considered MEC system. Moreover, letting (A13) divide by εS and making S→∞, we can get

(A24) $$\begin{array}{cc} \bar{Q}= & \lim_{S\to +\infty }\frac{1}{S}\sum_{t=0}^{S-1}\sum_{m\in M}\sum_{n\in N}Q_{m}^{n}\left(t\right)\leq \frac{c^{max}+V\left({\bar{e}}^{opt}-{\bar{e}}^{min}\right)}{\varepsilon }, \end{array}$$

where the inequality contained in (A24) follows from Assumption 1. Theorem 2 is eventually proved.
